# Association of Elevated Maternal Psychological Distress, Altered Fetal Brain, and Offspring Cognitive and Social-Emotional Outcomes at 18 Months

**DOI:** 10.1001/jamanetworkopen.2022.9244

**Published:** 2022-04-29

**Authors:** Yao Wu, Kristina M. Espinosa, Scott D. Barnett, Anushree Kapse, Jessica Lynn Quistorff, Catherine Lopez, Nickie Andescavage, Subechhya Pradhan, Yuan-Chiao Lu, Kushal Kapse, Diedtra Henderson, Gilbert Vezina, David Wessel, Adré J. du Plessis, Catherine Limperopoulos

**Affiliations:** 1 Developing Brain Institute, Children’s National Hospital, Washington, DC; 2 Division of Neonatology, Children’s National Hospital, Washington, DC; 3 Department of Diagnostic Imaging and Radiology, Children’s National Hospital, Washington, DC; 4 Hospital and Specialty Services, Children’s National Hospital, Washington, DC; 5 Prenatal Pediatrics Institute, Children’s National Hospital, Washington, DC

## Abstract

**Importance:**

Prenatal maternal psychological distress is associated with disturbances in fetal brain development. However, the association between altered fetal brain development, prenatal maternal psychological distress, and long-term neurodevelopmental outcomes is unknown.

**Objective:**

To determine the association of fetal brain development using 3-dimensional magnetic resonance imaging (MRI) volumes, cortical folding, and metabolites in the setting of maternal psychological distress with infant 18-month neurodevelopment.

**Design, Setting, and Participants:**

Healthy mother-infant dyads were prospectively recruited into a longitudinal observational cohort study from January 2016 to October 2020 at Children’s National Hospital in Washington, DC. Data analysis was performed from January 2016 to July 2021.

**Exposures:**

Prenatal maternal stress, anxiety, and depression.

**Main Outcomes and Measures:**

Prenatal maternal stress, anxiety, and depression were measured using validated self-report questionnaires. Fetal brain volumes and cortical folding were measured from 3-dimensional, reconstructed T2-weighted MRI scans. Fetal brain creatine and choline were quantified using proton magnetic resonance spectroscopy. Infant neurodevelopment at 18 months was measured using Bayley Scales of Infant and Toddler Development III and Infant-Toddler Social and Emotional Assessment. The parenting stress in the parent-child dyad was measured using the Parenting Stress Index-Short Form at 18-month testing.

**Results:**

The cohort consisted of 97 mother-infant dyads (mean [SD] maternal age, 34.79 [5.64] years) who underwent 184 fetal MRI visits (87 participants with 2 fetal studies each) with maternal psychological distress measures between 24 and 40 gestational weeks and completed follow-up infant neurodevelopmental testing. Prenatal maternal stress was negatively associated with infant cognitive performance (β = −0.51; 95% CI, −0.92 to −0.09; *P* = .01), and this association was mediated by fetal left hippocampal volume. In addition, prenatal maternal anxiety, stress, and depression were positively associated with all parenting stress measures at 18-month testing. Finally, fetal cortical local gyrification index and sulcal depth were negatively associated with infant social-emotional performance (local gyrification index: β = −54.62; 95% CI, −85.05 to −24.19; *P* < .001; sulcal depth: β = −14.22; 95% CI, −23.59 to −4.85; *P* = .002) and competence scores (local gyrification index: β = −24.01; 95% CI, −40.34 to −7.69; *P* = .003; sulcal depth: β = −7.53; 95% CI, −11.73 to −3.32; *P* < .001).

**Conclusions and Relevance:**

In this cohort study of 97 mother-infant dyads, fetal cortical local gyrification index and sulcal depth were associated with infant 18-month social-emotional and competence outcomes, and fetal left hippocampal volume mediated the association between prenatal maternal stress and infant cognitive outcome. These findings suggest that altered prenatal brain development in the setting of elevated maternal distress has adverse infant sociocognitive outcomes, and identifying early biomarkers associated with long-term neurodevelopment may assist in early targeted interventions.

## Key Points

**Question:**

Is altered fetal brain development in the setting of maternal psychological distress associated with infant neurodevelopment?

**Findings:**

In this cohort study of 97 mother-infant dyads who underwent 184 fetal magnetic resonance imaging studies (87 participants with 2 fetal studies each) and infant neurodevelopmental testing at 18 months, prenatal maternal stress was negatively associated with infant cognitive outcome, and this association was mediated by fetal left hippocampal volume. The study also found that increased fetal cortical local gyrification index and sulcal depth under elevated prenatal maternal distress were associated with decreased infant social-emotional scores measured by Bayley Scales of Infant and Toddler Development and competence scores measured by Infant-Toddler Social and Emotional Assessment.

**Meaning:**

These findings suggest that altered in vivo fetal brain development in the setting of elevated prenatal maternal psychological distress may be associated with adverse neurodevelopment.

## Teaser

This cohort study examines the association between infant 18-month neurodevelopment and fetal brain development using 3-dimensional magnetic resonance imaging volumes, cortical folding, and metabolites in the setting of maternal psychological distress.

## Introduction

Stress-related symptoms are now recognized as the most common complication of pregnancy, affecting approximately 1 of every 4 women, even those with healthy pregnancies and high socioeconomic status.^1^ Prenatal maternal stress exposure has been shown to have enduring consequences on brain development in the offspring, including altered regional brain volumetric growth (eg, amygdala, hippocampal, cerebellar, and cortical gray matter volumes), cortical folding, metabolism, microstructure, and functional connectivity,^1,2,3,4,5,6,7,8,9,10,11,12,13^ as well as long-term neurodevelopmental impairments (eg, cognitive, language, learning, and psychiatric dysfunctions).^14,15,16,17^

Neurodevelopmental problems in the setting of elevated maternal stress are thought to be associated with abnormal brain structure and circuitry.^18,19^ Notably, disaster-related prenatal maternal stress has been associated with larger amygdala volumes in children at 11 years old, and amygdala volume mediated the association between prenatal maternal stress and children’s externalizing problems.^20^ Similarly, maternal stress hormone (ie, cortisol) levels at early gestation have been associated with a larger right amygdala volume in girls at age 7 years, and amygdala volume mediated the association between prenatal maternal cortisol and children’s affective problems.^8^ In addition, prenatal maternal stress was associated with both reduced cortical thickness in children at age 7 years and elevated depressive symptoms at follow-up age 12 years, and children’s cortical thickness was associated with later depressive symptoms,^21^ suggesting a role of altered cortical thickness in the setting of prenatal maternal stress and adolescent depressive symptoms. Although a growing body of evidence links prenatal maternal stress exposure to altered brain growth and long-term neurodevelopment in the offspring, measures of in vivo fetal brain development using advanced magnetic resonance imaging (MRI) have been very limited.

Exploring in utero fetal brain development is challenging because of fetal and maternal motion, imaging artifacts, signal-to-noise ratio issues, changes in morphology (due to brain growth), and changes in image intensity (due to myelination and cortical maturation). Recently, advances in in utero MRI techniques^1,3,22,23^ enabled us to detect early alterations in fetal brain development under stress exposure, which may forecast later neurodevelopmental problems at an early age.^18^ In a previous study,^1^ we reported for the first time that maternal stress, anxiety, or depression, even if not reaching the severity of a mental disorder, was associated with adverse fetal brain development, including impaired fetal brain choline and creatine levels and hippocampal growth, as well as accelerated cortical folding. However, it is unknown whether and how these findings of altered in vivo fetal brain development are associated with subsequent infant neurodevelopment. In this study, we expand on our prior work^1^ by (1) examining the association between prenatal maternal psychological distress (ie, stress, anxiety, and depression that did not reach the severity of a mental disorder^1,24^) and infant 18-month neurodevelopment; (2) examining the association between fetal brain development (ie, volumetric, cortical folding, and metabolic measures) and infant 18-month neurodevelopment; and (3) determining whether fetal brain development mediates the association between prenatal maternal psychological distress and infant neurodevelopment outcomes.

## Methods

### Study Design

We prospectively recruited pregnant women and their fetuses into a longitudinal, observational cohort study between January 2016 and October 2020. Pregnant women were healthy volunteers from low-risk obstetric clinics. Women were eligible if medical record reviews confirmed a normal prenatal medical history, without chronic or pregnancy-induced illnesses, normal screening serum values, and normal fetal ultrasonography and fetal biometry studies. We excluded fetuses with known or suspected congenital infection, dysgenetic lesions or dysmorphic features, or genetic abnormalities. We also excluded pregnant women with (1) chronic medical conditions identified at the time of screening through the medical record or by self-report of existing medical conditions during each study visit (eg, autoimmune, genetic, metabolic, or psychiatric disorders); (2) pregnancy-related complications; (3) multiple pregnancies; (4) self-reported drug abuse, smoking, and alcohol use; (5) medications for chronic conditions; and (6) contraindications to MRI. Participants identified their race from a list of options defined on the basis of the information in US Census. Race was assessed in this study to help to understand underlying contributing factors. Fetal brain MRI studies were scheduled at 2 time points between 24 and 40 weeks of gestation.

This is a follow-up study of our previous research.^1^ Ninety-two of 97 participants (95%) in the current study were from our previous report^1^ who completed the follow-up infant 18-month neurodevelopmental testing. In our previous publication,^1^ we reported significant associations between maternal psychological distress and specific fetal MRI-based brain measures, including volumetric growth of the hippocampus, cortical folding metrics (local gyrification index and sulcal depth), and brain metabolic measures (creatine and choline levels). We now sought to determine the association between these fetal brain measures and infant neurodevelopmental outcomes at 18 months.

 This study was approved by the institutional review board at Children’s National Hospital, and written informed consent was obtained from all participants before enrollment. We followed the Strengthening the Reporting of Observational Studies in Epidemiology (STROBE) reporting guideline.

### Prenatal Maternal Psychological Distress

Psychometrically sound questionnaires measuring stress, anxiety, and depression were used and completed on the same day as each fetal MRI visit. The Perceived Stress Scale-10 (PSS-10, range, 0-40) assesses the self-reported level of stress in the prior month.^25^ Total scores of 15 or higher have been suggested as indicative of elevated maternal stress.^26,27^ The Spielberger State-Trait Anxiety Inventory is composed of 2 self-reported measures of anxiety: state anxiety (SSAI) and trait anxiety (STAI).^28^ Both measures are composed of 20 items (range, 20-80). SSAI and STAI scores of 40 or higher are suggestive of presence of anxiety.^29,30^ The Edinburgh Postnatal Depression Scale (EPDS; range, 0-30) screens depression symptoms in pregnant and postpartum women.^31,32^ EPDS scores of 10 or higher are suggestive of elevated depression.^33,34^ In this study, mothers who endorsed 1 or more measure of stress, anxiety, or depression (measured score greater than or equal to cutoff score) were referred to as positive for maternal psychological distress.

### MRI Acquisition and Processing

**Figure 1.**
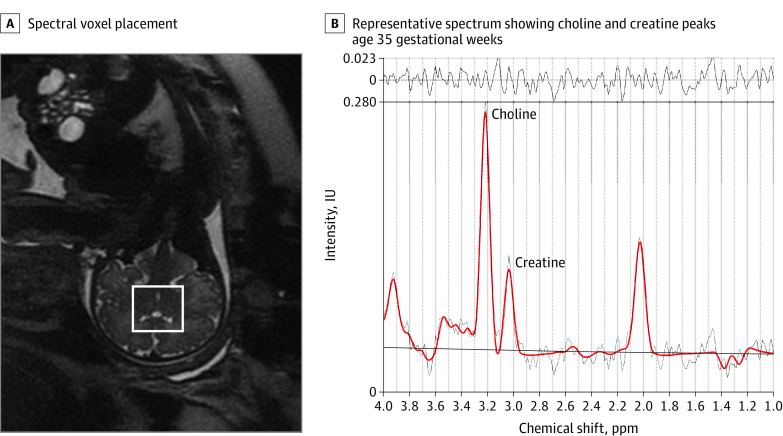
Fetal Brain Metabolic Measures A, The spectral voxel (square) was placed in the center of a fetal brain using the anatomical image as a guidance. B, Representative spectrum shows choline and creatine peaks (parts per million [ppm]) of a fetus at 35 gestational weeks. IU indicates international units.

As reported in our previous study,^1^ fetal brain T2-weighted MRI was performed using a 1.5-Tesla magnet (GE Discovery MR450) and an 8-channel receiver coil at 2 time points between 24 and 40 weeks of gestation. The scanning protocol included multiplanar, single-shot, fast spin-echo acquisitions (repetition time, 1100 ms; echo time, 160 ms; flip angle, 90°; field of view, 32 cm; matrix, 256 × 192; 2-mm slice thickness). The acquisition time was 2 to 3 minutes for each plane. Participants were free-breathing during the scan. After acquiring the 2-dimensional (2D) brain slices from axial, sagittal, and coronal planes, a state-of-the-art motion correction technique^35^ that has been previously validated was used to correct the fetal brain motion and reconstruct the images from 2D slices of all 3 planes to a high-resolution 3-dimensional (3D) image (eFigure 1 in the Supplement). After that, the reconstructed brains were spatially oriented to the brain atlas^36^ using landmark-based rigid registration in the IRTK package.^37^ The oriented images with the resolution of 0.86 × 0.86 × 0.86 mm^3^ were used to measure brain volume and cortical folding. For ^1^H–magnetic resonance spectroscopy (^1^H-MRS), a spectral voxel was placed in the center of the fetal brain to measure brain metabolites (Figure 1). More details on ^1^H-MRS acquisition have been reported in our previous study.^1^

### Brain Volume, Cortical Folding, and Metabolism

**Figure 2.**
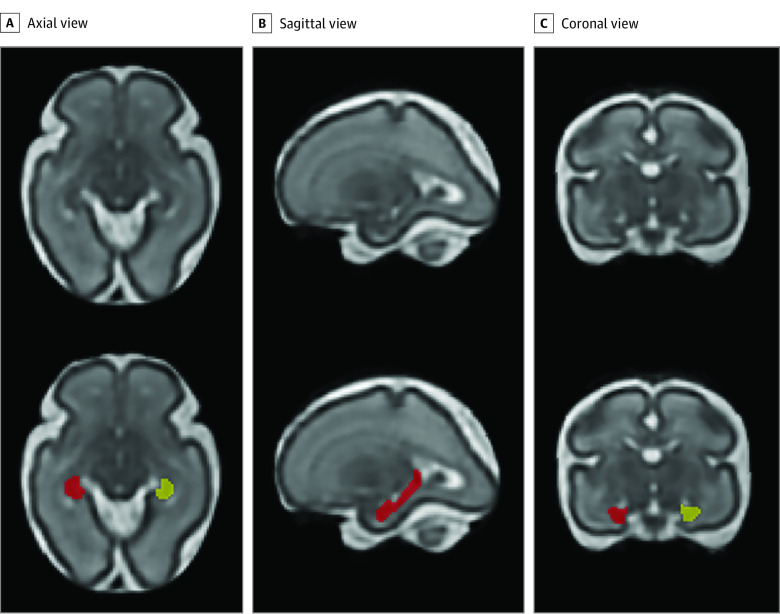
Segmentation of the Hippocampus Left hippocampus (yellow) and right hippocampus (red) are shown on a 3-dimensional reconstructed T2-weighted magnetic resonance image of a fetus at 27.9 gestational weeks.

Left and right hippocampi were manually delineated on reconstructed T2-weighted MR images (Figure 2) according to previously validated anatomical criteria.^38,39^ An experienced neuroradiologist (G.V.) assisted with anatomical localization of the hippocampi on MR images. The manual segmentation was performed by the same rater (Y.W.), and 20% of scans were randomly selected and segmented by another experienced rater (K.K.). Interrater reliabilities using intraclass correlation coefficient were higher than 0.95. The cortical folding measures, including local gyrification index^40^ and sulcal depth,^41^ were calculated on the inner surface of cortical gray matter.^1^ The local gyrification index was calculated as the ratio between the cortical surface area at each vertex and the corresponding area on the cerebral hull surface,^40^ and sulcal depth was measured as the distance from each vertex on the cortical surface to the nearest point on the cerebral hull surface.^41^ For ^1^H-MRS data, creatine and choline measured with Cramer-Rao lower bounds less than 20% from the fetal brain were used in the analysis.^1^

### Infant Neurodevelopmental Testing

Measures of infant 18-month neurodevelopmental status were performed by an examiner (K.E.) blinded to clinical findings. Neurodevelopmental assessments were conducted using the Bayley Scales of Infant and Toddler Development, Third Edition (BSID-III)^42^ and the Infant-Toddler Social and Emotional Assessment (ITSEA).^43^ The BSID-III evaluates cognitive, language, and motor functioning and was performed by a licensed psychologist (K.E.). The BSID-III Social-Emotional Scale measures adaptive behavior and social-emotional development via parent-completed measure. The normalized mean (SD) of each composite score is 100 (15). A mean score less than 85 indicates a developmental delay.^44^ The ITSEA is a parent-completed measure that evaluates child social-emotional and/or behavioral problems and delays in competence.^45^ The ITSEA assesses 4 domains: externalizing (activity/impulsivity, aggression/defiance, and peer aggression), internalizing (depression/withdrawal, general anxiety, separation distress, and inhibition to novelty), dysregulation (negative emotionality, sleep, eating, and sensory sensitivity), and competence (compliance, attention, mastery, motivation, imitation/play, empathy, and prosocial peer relations).^43,46^ Externalizing, internalizing, dysregulation scores of 65 or higher and competence domain scores of 35 or lower indicate a deficit or delay.^46^

### Parenting Stress at 18 Months

The Parenting Stress Index–Short Form (PSI-SF)^47^ was used to evaluate the degree of stress in the parent-child dyad at 18-month testing. The PSI-SF is a self-reported measure of parenting stress that includes 3 subscales: parental distress, parent-child dysfunctional interaction, and difficult child.^47,48^ Each subscale (range, 12-60) consists of 12 items rated from 1 (strongly disagree) to 5 (strongly agree). The PSI-SF also gives a defensive responding scale (7 items from the parental distress scale; range, 7-35) and a total stress scale (range, 36-180) that is a sum of parental distress, parent-child dysfunctional interaction, and difficult child.^47,48^ The PSI-SF has been validated to be effective and appropriate for measuring stress in the parent-child system in a variety of populations.^49,50,51,52,53^

### Statistical Analysis

Analyses were performed using SAS statistical software version 9.3 (SAS Institute) and MATLAB statistical software version R2018b (MathWorks). Data analysis was performed from January 2016 to July 2021. Characteristics of the study sample in negative vs positive prenatal maternal psychological distress were compared using either *t* test or Fisher exact test, where appropriate. Infant neurodevelopmental scores in women positive on 1 or more prenatal distress measures were compared using analysis of covariance, controlling for possible confounders including maternal education and employment,^54^ PSI-SF total stress scale at 18 months, and neurodevelopmental assessment during COVID-19 pandemic (yes or no). Generalized estimating equations (GEEs), which allow multiple measurements for each participant, were used to measure associations between prenatal maternal psychological distress scores and infant neurodevelopmental outcomes, controlling for gestational age (GA) at the fetal visit, maternal education and employment, PSI-SF total stress scale, and neurodevelopmental assessment during the COVID-19 pandemic (yes or no). An unstructured correlation assumption was used to address the possible correlation within participants due to multiple measurements. GEE was used to determine the associations between prenatal maternal psychological distress scores and PSI-SF parenting stress scales measured at 18 months, controlling for GA at fetal visit, maternal education and employment, and neurodevelopmental assessment during COVID-19 pandemic (yes or no). GEE was also used to measure associations between fetal brain measures (volume, cortical folding, and metabolites) and infant neurodevelopmental outcomes, adjusting for prenatal maternal psychological distress status (positive or negative), GA at fetal visit, maternal education, maternal employment, PSI-SF total stress scale at 18 months, and neurodevelopmental assessment during COVID-19 pandemic (yes or no). The prenatal maternal psychological distress status was derived by combining the individual positive/negative distress measurements of participants (SSAI, STAI, PSS, and EPDS). In the combined measurement, participants were defined as positive if they had 1 or more positive individual distress measurements; otherwise, they were defined as negative. Additional adjustments for maternal body mass index, age, race, GA at birth, and birth weight were considered, with no material effect on estimates. The significance level of multiple testing was adjusted using the false discovery rate method,^55^ and adjusted 2-sided *P* ≤ .05 was considered significant. Causal mediation analyses^56^ were performed to assess whether fetal brain development mediated the association between prenatal maternal distress scores and infant neurodevelopment at 18 months. The direct effect of prenatal maternal distress on infant neurodevelopment and the indirect (mediating) effect of prenatal maternal distress on infant neurodevelopment through fetal brain volumes were evaluated using the following 3 models: model 1, *Y* = *i*_1_ + β_1_*X* + γ_1_C_1_ + *e*_1_, where β_1_ is the total effect of *X* on *Y*; model 2, *M* = *i*_2_+ β_2_*X* + γ_2_*C*_2_ + *e*_2_, where β_2_ tests the association between *X* and the mediator *M*; and model 3, *Y* = *i*_3_ + β_3_*X* + β_4_*M* + γ_3_*C*_1_+*e*_3_, where β_3_ is the direct effect of *X* on *Y* (*X* = prenatal maternal distress score, *Y* = infant neurodevelopment outcome, and *M* = fetal brain volume). *C*_1_ denotes the vector of confounding factors that include GA at the fetal visit, maternal education, maternal employment, PSI-SF total stress scale at 18 months, and neurodevelopmental assessment during COVID-19 pandemic (yes or no). *C*_2_ is a vector of confounding factors in model 2.^1^ If *X* and *M* and have no association in model 2, there is no ground for mediation. The mediating effect of *X* on *Y* through mediator *M* is the total effect in model 1 minus the direct effect in model 3 (β_1 _− β_3_). Bootstrapping (1000 samples) was used to estimate bias-corrected 95% CIs, and a significant mediating effect was defined as a bootstrapped 95% CI that did not include 0.

## Results

### Demographic Data

Our cohort consisted of 97 mother-fetus dyads (49 [51%] male fetuses and 48 [49%] female fetuses) who underwent 184 fetal MRI visits (87 participants with 2 fetal studies each) with maternal psychological distress measures between 24 and 40 gestational weeks and completed follow-up infant neurodevelopmental testing at 18 months. We initially enrolled 131 study participants; however, 1 participant was excluded because of an abnormal fetal MRI finding (ie, intraventricular hemorrhage). For the remaining participants, 101 completed both the prenatal MRI visits and follow-up infant neurodevelopmental testing. Four of 101 participants with missing prenatal maternal distress questionnaires were excluded. Ten of the remaining 97 participants completed the first but not the second MRI visit, resulting in 184 fetal MRI studies included in the analyses. Data from 13% of T2-weighted scans and 17% of ^1^H-MRS were not usable because of fetal motion. For T2-weighted MRI scans, left and right hippocampi were successfully measured on 92 fetuses (153 scans), and cortical folding was successfully measured on 80 fetuses (117 scans). For ^1^H-MRS scans, fetal brain choline and creatine levels were successfully analyzed on 84 fetuses (153 scans). All conventional fetal MRI scans were interpreted as structurally normal by an experienced fetal neuroradiologist (G.V.). The conventional MRI findings were shared with the study participants. The mean (SD) GA was 28.12 (2.41) weeks at the first fetal MRI and 35.95 (1.70) weeks at the second fetal MRI. The mean (SD) age at neurodevelopment testing was 19.68 (4.54) months. The mean (SD) maternal age was 34.79 (5.64) years. Ninety-one women (94%) attended college and 84 (87%) had professional employment. For maternal race, 7 women (7%) were Asian/Pacific Islander, 10 (10%) were Hispanic, 15 (15%) were non-Hispanic Black, and 60 (62%) were non-Hispanic White. All fetal MRI and prenatal maternal distress measures were performed before May 2019. Fourteen of our 97 participants completed their infant neurodevelopmental testing since the first case of COVID-19 was reported in the US on January 20, 2020. Participant characteristics are summarized in Table 1.

**Table 1.** Characteristics of the Overall Study Sample and by Prenatal Maternal Psychological Distress Status

Variable	Patients, No. (%)			*P* value^a^
	Overall (N = 97 [100%])	Negative (n = 62 [64%])	Positive (n = 35 [36%])	
Age, mean (SD)				
At fetal visit 1, wk (n = 97)	28.12 (2.41)	28.10 (2.55)	28.20 (2.27)	.84
At fetal visit 2, wk (n = 87)	35.95 (1.70)	35.94 (1.75)	35.99 (1.61)	.88
At neurodevelopment testing, mo (n = 97)	19.68 (4.54)	19.84 (4.50)	19.39 (4.66)	.64
Birth measures, mean (SD)				
Age, wk	39.16 (1.41)	39.09 (1.52)	39.29 (1.19)	.49
Weight, g	3259 (512)	3267 (518)	3246 (510)	.84
Characteristics				
Maternal age, mean (SD), y	34.79 (5.64)	35.18 (5.64)	34.09 (5.65)	.37
Maternal body mass index, mean (SD)^b^				
Fetal visit 1	46.19 (7.98)	46.30 (8.63)	45.66 (6.64)	.69
Fetal visit 2	48.20 (7.68)	48.14 (8.13)	48.32 (6.69)	.91
Primigravida	38 (39)	26 (42)	12 (34)	.30
Primipara	51 (53)	35 (56)	16 (46)	.51
Parental education				
Partial high school				.66 for mother; .12 for father
Maternal	0	0	0	
Paternal	1 (1)	0	1 (3)	
High school				
Maternal	3 (3)	2 (3)	1 (3)	
Paternal	9 (9)	5 (8)	4 (11)	
Partial college				
Maternal	8 (8)	4 (6)	4 (11)	
Paternal	12 (12)	11 (18)	1 (3)	
College graduate				
Maternal	26 (27)	19 (31)	7 (20)	
Paternal	23 (24)	13 (21)	10 (29)	
Graduate degree				
Maternal	57 (59)	36 (58)	21 (60)	
Paternal	47 (48)	30 (48)	17 (49)	
Unknown				
Maternal	3 (3)	1 (2)	2 (6)	
Paternal	5 (5)	3 (5)	2 (6)	
Parental employment				
Professional				.18 for mother; .86 for father
Maternal	84 (87)	55 (89)	29 (83)	
Paternal	77 (79)	51 (82)	26 (74)	
Skilled/clerical/sales				
Maternal	3 (3)	3 (5)	0	
Paternal	5 (5)	3 (5)	2 (6)	
Semiskilled operator				
Maternal	2 (2)	0	2 (6)	
Paternal	3 (3)	2 (3)	1 (3)	
Unemployed/homemaker				
Maternal	5 (5)	3 (5)	2 (6)	
Paternal	6 (6)	3 (5)	3 (9)	
Unknown				
Maternal	3 (3)	1 (2)	2 (6)	
Paternal	6 (6)	3 (5)	3 (9)	
Maternal race and ethnicity				
American Indian or Alaskan Indian	0	0	0	.80
Asian/Pacific Islander	7 (7)	5 (8)	2 (6)	
Hispanic	10 (10)	8 (13)	2 (6)	
Non-Hispanic Black	15 (15)	9 (15)	6 (17)	
Non-Hispanic White	60 (62)	38 (61)	22 (63)	
Other or unknown^c^	5 (5)	2 (3)	3 (9)	
Distribution of endorsed maternal psychological distress				
1	12 (12)	NA	12 (34)	NA
2	8 (8)	NA	8 (23)	
3	9 (9)	NA	9 (26)	
4	6 (6)	NA	6 (17)	

Abbreviation: NA, not applicable.

^a^ *P* value for difference between participants with negative and positive maternal psychological distress based on *t* test for continuous variables and Fisher exact test for categorical variables.

^b^ Body mass index is calculated as weight in kilograms divided by height in meters squared.

^c^ Other is a self-reported option that includes all other responses not included in any other race category.

### Prenatal Maternal Psychological Distress Measures

Mean (SD) maternal anxiety, stress, and depression scores were 29.6 (8.9) for SSAI, 30.7 (8.6) for STAI, 10.7 (5.8) for PSS, and 4.3 (3.8) for EPDS. The distress scores across GA are shown in eFigure 2 in the Supplement. Of the 97 pregnant women, 28 (29%) were positive (measured score greater than or equal to the cutoff score) for stress, 29 (30%) for anxiety (22 [23%] for state anxiety and 19 [20%] for trait anxiety), and 10 (10%) for depression. Thirty-five women (36%) tested positive for stress, anxiety, or depression. For the 35 women who tested positive on distress measures (ie, SSAI, STAI, PSS, or EPDS), 12 (34%) of them were positive on 1 distress measure, 8 (23%) on 2 measures, 9 (26%) on 3 measures, and 6 (17%) on all 4 measures (Table 1).

### Fetal Brain Volumes, Cortical Folding, and Metabolites

Mean (SD) volumes of left and right hippocampi were 0.52 (0.18) cm^3^ and 0.56 (0.18) cm^3^, respectively. Mean (SD) values of cortical folding index and sulcal depth were 1.29 (0.20) and 1.62 (0.63) mm, respectively. Mean (SD) fetal brain choline and creatine levels were 2.39 (0.34) IU and 2.94 (0.68) IU, respectively.

### Infant Neurodevelopmental Results and Parenting Stress Scales

The mean (SD) scores for the BSID-III were 108 (16) for cognitive, 102 (13) for language, 105 (10) for motor, 103 (15) for adaptive, and 113 (21) for social-emotional domains. Of the 97 infants, delayed development (score <85) was seen in 3 infants (3%) for cognitive, 6 infants (6%) for language, 1 infant (1%) for motor, 8 infants (8%) for adaptive, and 6 infants (6%) for social-emotional domains. The mean (SD) scores for ITSEA were 48 (8) for externalizing, 46 (10) for internalizing, 41 (11) for dysregulation, and 51 (10) for competence domains. Of the 97 infants, 4 (4%) had externalizing problems, 3 (3%) had internalizing problems, and 2 (2%) had dysregulation problems (measured score ≥65); 3 infants (3%) had delays in the competence domain (measured score ≤35). The mean (SD) scores for PSI-SF were 12 (4) for defensive responding, 21 (7) for parental distress, 16 (5) for parent-child dysfunctional interaction, 20 (6) for difficult child, and 56 (16) for total stress domains.

### Associations Between Prenatal Maternal Psychological Distress and Infant Neurodevelopmental Outcomes

Prenatal maternal stress was negatively associated with infant cognitive performance (β = −0.51; 95% CI, −0.92 to −0.09; *P* = .01) (Table 2). We further assessed whether infant neurodevelopment outcomes would differ in participants who tested positive on 1 vs multiple prenatal distress measures (SSAI, STAI, PSS, and/or EPDS). There was no significant difference in neurodevelopment outcomes among infants born to women who were positive on 1 vs multiple distress measures (eTable 1 in the Supplement). Therefore, we combined the participants with any (≥1) positive distress measure and referred to them as positive for maternal psychological distress.

**Table 2.** Regression Estimates for the Association of Prenatal Maternal Psychological Distress on Infant Neurodevelopment Outcome and Parenting Stress Index at 18-Month Testing^a^

Test domain	SSAI			STAI			PSS			EPDS		
	β (95% CI)	*P* value	*q*	β (95% CI)	*P* value	*q*	β (95% CI)	*P* value	*q*	β (95% CI)	*P* value	*q*
Infant neurodevelopment outcome^b^												
BSID-III												
Cognitive	−0.08 (0.22 to 0.07)	.30	.48	−0.27 (−0.56 to 0.01)	.06	.16	−0.51 (−0.92 to −0.09)	.01	.03	−0.26 (−0.56 to 0.05)	.10	.23
Language	0.004 (−0.11 to 0.11)	.94	.94	−0.04 (−0.25 to 0.17)	.70	.72	−0.05 (−0.33 to 0.22)	.69	.72	0.15 (−0.14 to 0.43)	.30	.48
Motor	−0.03 (−0.13 to 0.07)	.59	.68	−0.14 (−0.31 to 0.02)	.09	.21	−0.20 (−0.43 to 0.04)	.09	.21	−0.17 (−0.4 to 0.06)	.15	.31
Social-emotional	0.10 (−0.06 to 0.26)	.20	.37	0.07 (−0.18 to 0.33)	.56	.67	0.04 (−0.33 to 0.41)	.82	.83	−0.15 (−0.62 to 0.31)	.51	.64
Adaptive	0.08 (−0.08 to 0.23)	.33	.50	0.07 (−0.18 to 0.32)	.60	.68	−0.06 (−0.37 to 0.25)	.70	.72	0.12 (−0.31 to 0.55)	.57	.67
ITSEA												
Externalizing	0.01 (−0.03 to 0.06)	.48	.64	0.04 (−0.05 to 0.13)	.35	.50	0.04 (−0.06 to 0.15)	.43	.60	0.05 (−0.05 to 0.14)	.34	.50
Internalizing	0.07 (−0.03 to 0.18)	.17	.34	0.08 (−0.07 to 0.22)	.29	.48	0.16 (−0.05 to 0.37)	.13	.29	0.14 (−0.16 to 0.45)	.35	.50
Dysregulation	0.04 (−0.14 to 0.22)	.68	.72	0.13 (−0.09 to 0.36)	.24	.42	0.21 (−0.07 to 0.49)	.14	.30	0.15 (−0.29 to 0.59)	.50	.64
Competence	−0.03 (−0.12 to 0.06)	.45	.61	−0.05 (−0.2 to 0.11)	.53	.65	−0.05 (−0.24 to 0.15)	.63	.70	−0.14 (−0.39 to 0.1)	.24	.42
Parenting stress index at 18 mo^c^												
PSI-SF												
Defensive responding	0.07 (0.02 to 0.11)	.004	.01	0.15 (0.08 to 0.23)	<.001	<.001	0.20 (0.1 to 0.31)	<.001	<.001	0.13 (0.05 to 0.21)	.001	.005
Parental distress	0.12 (0.04 to 0.2)	.002	.008	0.26 (0.15 to 0.38)	<.001	<.001	0.35 (0.18 to 0.52)	<.001	<.001	0.28 (0.14 to 0.42)	<.001	<.001
Parent-child dysfunction interaction	0.05 (0.0006 to 0.09)	.04	.11	0.10 (0.03 to 0.17)	.002	.008	0.13 (0.05 to 0.22)	.002	.008	0.22 (0.1 to 0.34)	<.001	.001
Difficult child	0.05 (−0.03 to 0.12)	.20	.37	0.13 (0.02 to 0.24)	.02	.05	0.16 (0.02 to 0.31)	.02	.05	0.18 (0.04 to 0.32)	.01	.03
Total stress	0.22 (0.05 to 0.39)	.009	.03	0.49 (0.24 to 0.74)	<.001	<.001	0.65 (0.29 to 1.01)	<.001	.002	0.70 (0.35 to 1.05)	<.001	<.001

Abbreviations: BSID-III, Bayley Scales of Infant and Toddler Development, third edition; EPDS, Edinburgh Postnatal Depression Scale; ITSEA, Infant-Toddler Social and Emotional Assessment; PSI-SF, Parenting Stress Index-Short Form; PSS, Perceived Stress Scale; SSAI, Spielberger State Anxiety Inventory; STAI, Spielberger Trait Anxiety Inventory.

^a^ β is unstandardized coefficient that denotes the association for a 1-unit increase in prenatal maternal psychological distress scale and infant neurodevelopment outcome or parenting stress scale with 95% CIs around the estimate. *q* denotes adjusted *P* value using the false discovery rate; 56 tests were performed in this analysis.

^b^ Results based on generalized estimating equation models after controlling for gestational age at fetal visit, maternal education, maternal employment, total stress scale from PSI-SF at 18-month testing, and neurodevelopmental assessment during COVID-19 pandemic (yes or no).

^c^ Results based on generalized estimating equation models after controlling for gestational age at fetal visit, maternal education, maternal employment, and neurodevelopmental assessment during COVID-19 pandemic (yes or no).

### Associations Between Prenatal Maternal Psychological Distress and Parenting Stress at 18-Month Testing

Prenatal maternal trait anxiety, stress, and depression were positively associated with all PSI-SF scales at 18-month testing (Table 2). Significant associations between prenatal distress and PSI-SF outcomes were noted in defensive responding (STAI: β = 0.15; 95% CI, 0.08 to 0.23; PSS: β = 0.20; 95% CI, 0.1 to 0.31; EPDS: β = 0.13; 95% CI, 0.05 to 0.21), parental distress (STAI: β = 0.26; 95% CI, 0.15 to 0.38; PSS: β = 0.35; 95% CI, 0.18 to 0.52; EPDS: β = 0.28; 95% CI, 0.14 to 0.42), parental-child dysfunction interaction (EPDS: β = 0.22; 95% CI, 0.1 to 0.34), and total stress (STAI: β = 0.49; 95% CI, 0.24 to 0.74; PSS: β = 0.65; 95% CI, 0.29 to 1.01; EPDS: β = 0.70; 95% CI, 0.35 to 1.05) (*P* < .001 for all).

### Associations Between Fetal Brain Measures and Infant Neurodevelopmental Outcomes

Fetal cortical local gyrification index and sulcal depth were negatively associated with infant social-emotional performance (local gyrification index: β = −54.62; 95% CI, −85.05 to −24.19; *P* < .001; sulcal depth: β = −14.22; 95% CI, −23.59 to −4.85; *P* = .002) and competence scores (local gyrification index: β = −24.01; 95% CI, −40.34 to −7.69; *P* = .003; sulcal depth: β = −7.53, 95% CI, −11.73 to −3.32; *P* < .001) (Table 3). For fetal brain metabolic measures, choline and creatine levels were positively associated with infant adaptive behaviors (choline: β = 2.60; 95% CI, 0.40 to 4.79; *P* = .02; creatine: β = 1.58; 95% CI, 0.08 to 3.08; *P* = .03) (Table 3), but these associations were no longer significant after adjusting for multiple testing. Because prenatal maternal stress was negatively associated with infant cognitive performance (Table 2), we performed causal mediation analysis to measure whether this association was mediated by any fetal brain measure. We found that fetal left hippocampal volume accounted for 11% of the total prenatal maternal stress and infant cognitive outcome association (β = −0.11; bootstrapped 95% CI, −0.35 to −0.0002) (eTable 2 in the Supplement).

**Table 3.** Regression Estimates for the Association of Fetal Brain Measures on Infant Neurodevelopmental Scores^a^

Test domain	Volume						Cortical folding						Metabolites					
	Left hippocampus			Right hippocampus			Local gyrification index			Sulcal depth			Choline			Creatine		
	β (95% CI)	*P* value	*q*	β (95% CI)	*P* value	*q*	β (95% CI)	*P* value	*q*	β (95% CI)	*P* value	*q*	β (95% CI)	*P* value	*q*	β (95% CI)	*P* value	*q*
BSID-III																		
Cognitive	26.69 (−12.64 to 66.02)	.18	.66	4.15 (−26.57 to 34.87)	.79	.95	−2.02 (−23.74 to 19.70)	.85	.95	−0.11 (−6.38 to 6.16)	.97	.97	−0.24 (−2.22 to 1.74)	.81	.95	−0.42 (−1.57 to 0.74)	.47	.84
Language	−5.03 (−32.04 to 21.98)	.71	.95	−18.24 (−43.94 to 7.46)	.16	.66	−10.79 (−29.67 to 8.08)	.26	.66	−2.47 (−8.24 to 3.30)	.40	.77	0.11 (−1.66 to 1.87)	.90	.97	−1.28 (−2.67 to 0.10)	.06	.46
Motor	−4.05 (−29.15 to 21.04)	.75	.95	−13.39 (−37.76 to 10.97)	.28	.66	−2.22 (−21.14 to 16.71)	.81	.95	−1.45 (−7.05 to 4.15)	.61	.95	0.88 (−1.21 to 2.98)	.40	.77	−0.57 (−1.83 to 0.69)	.37	.76
Social-emotional	−10.88 (−59.22 to 37.47)	.65	.95	4.96 (−37.27 to 47.18)	.81	.95	−54.62 (−85.05 to −24.19)	.0004	.01	−14.22 (−23.59 to −4.85)	.002	.03	2.09 (−1.95 to 6.13)	.31	.66	1.90 (−0.21 to 4.01)	.07	.47
Adaptive	19.18 (−12.96 to 51.33)	.24	.66	3.77 (−25.18 to 32.71)	.79	.95	2.75 (−24.23 to 29.72)	.84	.95	0.33 (−7.60 to 8.25)	.93	.97	2.60 (0.40 to 4.79)	.02	.21	1.58 (0.08 to 3.08)	.03	.27
ITSEA																		
Externalizing	0.29 (−13.99 to 14.56)	.96	.97	6.69 (−6.40 to 19.78)	.31	.66	−9.11 (−23.26 to 5.05)	.20	.66	−3.09 (−7.18 to 1.0)	.13	.63	−0.07 (−0.83 to 0.69)	.85	.95	−0.27 (−0.78 to 0.23)	.29	.66
Internalizing	−11.44 (−29.68 to 6.80)	.21	.66	−1.29 (−17.20 to 14.62)	.87	.95	−8.19 (−23.91 to 7.54)	.30	.66	−1.30 (−5.25 to 2.65)	.52	.90	−0.22 (−0.92 to 0.49)	.54	.91	0.08 (−0.40 to 0.56)	.74	.95
Dysregulation	−12.41 (−31.89 to 7.07)	.21	.66	−2.54 (−21.73 to 16.65)	.79	.95	−13.10 (−28.76 to 2.55)	.10	.60	−3.10 (−7.20 to 1.0)	.13	.63	0.50 (−0.44 to 1.44)	.29	.66	0.26 (−0.38 to 0.90)	.42	.78
Competence	−0.57 (−20.28 to 19.14)	.95	.97	−10.46 (−30.78 to 9.85)	.31	.66	−24.01 (−40.34 to −7.69)	.003	.04	−7.53 (−11.73 to −3.32)	.0004	.01	0.23 (−0.79 to 1.24)	.66	.95	−0.14 (−0.77 to 0.49)	.65	.95

Abbreviations: BSID-III, Bayley Scales of Infant and Toddler Development, third edition; ITSEA, Infant-Toddler Social and Emotional Assessment.

^a^ Results are based on generalized estimating equation models controlling for gestational age at fetal visit, prenatal maternal psychological distress status (positive or negative), maternal education, maternal employment, total stress scale from Parenting Stress Index–Short Form at 18-month testing, and neurodevelopmental assessment during COVID-19 pandemic (yes or no). β is an unstandardized coefficient that denotes the association for a 1-unit increase in fetal brain measure and infant neurodevelopment outcome with 95% CIs around the estimate. *q* denotes adjusted *P* value using the false discovery rate; 54 tests were performed in this analysis.

## Discussion

In this longitudinal, prospective, observational cohort study, we found that prenatal maternal stress was inversely associated with infant cognitive outcome, and this association was mediated by fetal left hippocampal volume. In addition, prenatal maternal stress, anxiety, and depression were positively associated with parenting stress scores at 18-month testing. To our knowledge, this is the first study to demonstrate that altered human fetal cortical folding may be associated with infant neurodevelopment. Specifically, fetal cortical local gyrification index and sulcal depth were negatively associated with infant social-emotional and competence performance.

The exact incidence of mental health disturbances in pregnant women is not known but is likely underestimated. In this study, all pregnant participants were healthy and had low-risk pregnancies, most were well educated and employed, and most were living in areas (Washington, DC) with good access to health care. Despite these seemingly favorable conditions, 36% of participants exceeded the positivity threshold for stress, anxiety, and/or depression. This is in keeping with recent studies^57,58,59^ reporting prenatal depression and anxiety in up to 18.4% and 25.3%, respectively, of women in high-income countries and of middle-to-high socioeconomic status. Furthermore, we found that prenatal maternal stress, depression, and anxiety were correlated with all PSI-SF scores at 18-month testing. This finding suggests that prenatal maternal distress may not be transient but persistent across the postnatal period with subsequent influences on both the parent-child interaction and infant self-regulation.

In addition, we found that prenatal maternal stress, even if not reaching the severity of a mental disorder, was associated with decreased infant cognitive performance. This finding is in keeping with results of previous studies^14,15,16,60,61^ showing cognitive impairments in children following early exposure to maternal stress. In particular, our findings suggest that this association may be partially mediated by fetal left hippocampal volume. This is supported by our previous study that maternal stress decreased fetal left hippocampal volume,^1^ as well as other studies that the hippocampal subregions were related to certain cognitive functions.^62,63^ The standardized assessment of infant cognitive performance further supports this mediation finding. Our bilateral asymmetrical finding may be explained by different functions and growth rates of left and right hippocampus.^1,38,64^ Our findings suggest that although the prevalence of prenatal maternal distress in our cohort may not be as high as in the high-risk population,^3,65^ its association with infant outcomes cannot be ignored.

Importantly, we found that increased fetal cortical gyrification index and sulcal depth were associated with decreased infant social-emotional and competence performance. Increased cortical gyrification has been suggested in children with dyslexia and autism,^66,67^ and sulcal depth has been associated with the severity of impaired performance on working memory and executive function in adults with schizophrenia.^68^ Our earlier study suggested that prenatal maternal psychological distress increased fetal cortical gyrification index and sulcal depth.^1^ The current study extends our previous findings and suggests a critical role for disturbances in emerging fetal cerebral cortical folding development in mediating the association between prenatal maternal distress and neurodevelopmental problems that later manifest in infancy.

In addition, we found positive associations between fetal brain choline and creatine levels and infant adaptive behaviors, although these associations were not significant after adjusting for multiple testing. Animal studies^69^ have suggested that there are associations between choline status and attention and memory, and choline supplementation during pregnancy improves cognitive and neurological function in offspring. In human studies,^70^ maternal plasma choline level in the second trimester was positively associated with cognitive development in 18-month-old infants. Levels of brain creatine have been linked to cognitive and emotional processing in infancy, and alterations in the brain creatine pathway have been related to psychiatric disorders.^71^ In addition, brain metabolites in healthy neonates have been associated with learning and memory in infants at 4 months.^72^ Our current study suggests that increased in utero fetal brain choline and creatine levels, in the setting of decreased prenatal maternal depression and stress reported in our previous study,^1^ are likely associated with better infant adaptive performance, which needs to be confirmed in a larger cohort.

These findings are particularly insightful, given the nature of the ongoing COVID-19 pandemic; reports of increased maternal anxiety, stress, and depression^73,74^; and the underexplored nexus between elevated maternal distress during the pandemic and the health of the next generation of infants. More than 1 million US infants have been born in the COVID-19 pandemic era, yet we lack knowledge about the influence of pandemic-related maternal distress on infants’ long-term neurodevelopment. Our ongoing studies will continue to explore the association between heightened maternal distress amid the pandemic and children’s lifelong health.

### Limitations

This study has potential limitations. First, maternal distress assessment at certain time points may not fully reflect maternal mental health status for the entire pregnancy. Second, the percentage of women positive for stress, anxiety, and depression will change using different cutoff scores. We chose cutoffs used for pregnant women in both our earlier work^1,3^ and those of others,^26,27,29,30,33,34^ but we acknowledge the potential for either false positives or false negatives. Third, in this study, cognitive, language, and motor skills on the Bayley Scales were evaluated by a licensed psychologist. However, infant social-emotional assessments and prenatal maternal psychological distress measures were based on maternal report. Although these maternal questionnaires are widely used in the literature and standardized with established psychometric properties, we acknowledge the possible bias that may exist in parent-reported measures. Fourth, data from 13% of T2-weighted scans and 17% of ^1^H-MRS images were not usable because of fetal motion; however, our percentage of lost data is similar to that in other fetal studies.^75,76^ Fifth, participants in our catchment area may not be reflective of other regions. The metropolitan Washington, DC, area is home to a highly educated, low-unemployment workforce, which may have increased access to health care needs not reflective of other geographical areas.

## Conclusions

In conclusion, we report that prenatal maternal stress is associated with infant cognitive outcome, and this association is partially mediated by fetal left hippocampal volume. In addition, we report that increased fetal cortical gyrification index and sulcal depth in pregnancies complicated by maternal psychological distress is associated with decreased infant social-emotional and competence performance. Identifying early brain developmental biomarkers may help improve the identification of infants at risk for later neurodevelopmental impairment who might benefit from early targeted interventions.

## Notes

Supplement. **eFigure 1. **An Example of Fetal Brain Reconstruction From 2D Single Shot Fast Spin Echo MRI Slices of Coronal, Sagittal, and Axial Planes (Left) to a Single 3D Volume (Right) **eFigure 2.** Prenatal Maternal State Anxiety (SSAI), Trait Anxiety (STAI), Stress (PSS), and Depression (EPDS) Scores Across Gestational Age (Weeks) **eTable 1.** Infant Neurodevelopmental Outcomes With One and More Positive Maternal Distress Measures (SSAI, STAI, PSS, and/or EPDS) **eTable 2.** Causal Mediation Analysis of the Relationship Between Prenatal Maternal Stress and 18-Month Cognitive Outcome With Fetal Brain Measure as the Mediator
