# Supplementary material for: Association of Elevated Maternal Psychological Distress, Altered Fetal Brain, and Offspring Cognitive and Social-Emotional Outcomes at 18 Months
Source: JAMA Netw Open. 2022 Apr 29;5(4):e229244. doi: 10.1001/jamanetworkopen.2022.9244 (PMC9055453; doi:10.1001/jamanetworkopen.2022.9244)
Supplement: Supplement. — eFigure 1. An Example of Fetal Brain Reconstruction From 2D Single Shot Fast Spin Echo MRI Slices of Coronal, Sagittal, and Axial Planes (Left) to a Single 3D Volume (Right) eFigure 2. Prenatal Maternal State Anxiety (SSAI), Trait Anxiety (STAI), Stress (PSS), and Depression (EPDS) Scores Across Gestational Age (Weeks) eTable 1. Infant Neurodevelopmental Outcomes With One and More Positive Maternal Distress Measures (SSAI, STAI, PSS, and/or EPDS) eTable 2. Causal Mediation Analysis of the Relationship Between Prenatal Maternal Stress and 18-Month Cognitive Outcome With Fetal Brain Measure as the Mediator [file jamanetwopen-e229244-s001.pdf]

Supplemental Online Content

Wu Y, Espinosa KM, Barnett SD, et al. Association of elevated maternal psychological distress, altered fetal brain, and offspring cognitive and social-emotional outcomes at 18 months. *JAMA Netw Open*. 2022;5(4):e229244. doi:10.1001/jamanetworkopen.2022.9244

eFigure 1. An Example of Fetal Brain Reconstruction From 2D Single Shot Fast Spin Echo MRI Slices of Coronal, Sagittal, and Axial Planes (Left) to a Single 3D Volume (Right)

eFigure 2. Prenatal Maternal State Anxiety (SSAI), Trait Anxiety (STAI), Stress (PSS), and Depression (EPDS) Scores Across Gestational Age (Weeks)

eTable 1. Infant Neurodevelopmental Outcomes With One and More Positive Maternal Distress Measures (SSAI, STAI, PSS, and/or EPDS)

eTable 2. Causal Mediation Analysis of the Relationship Between Prenatal Maternal Stress and 18-Month Cognitive Outcome With Fetal Brain Measure as the Mediator

This supplemental material has been provided by the authors to give readers additional information about their work.

© 2022 Wu Y et al. *JAMA Network Open.*

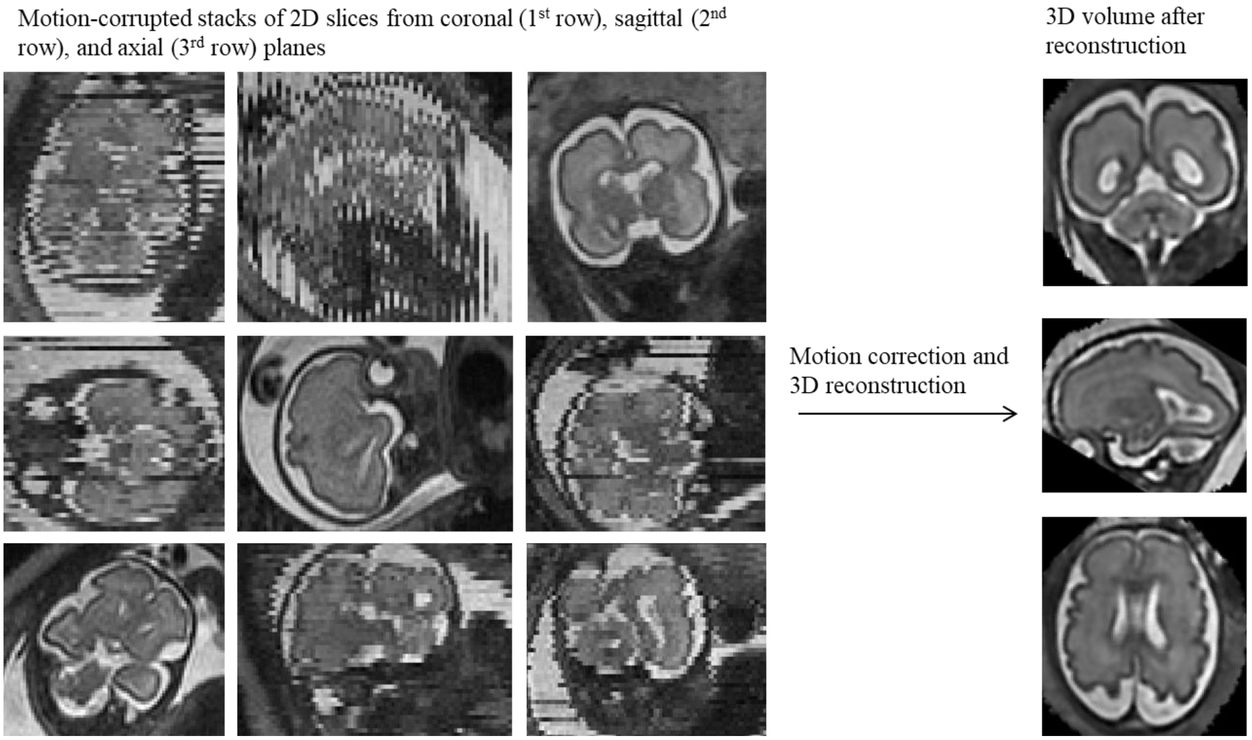

eFigure 1. An Example of Fetal Brain Reconstruction From 2D Single Shot Fast Spin Echo MRI Slices of Coronal, Sagittal, and Axial Planes (Left) to a Single 3D Volume (Right)

© 2022 Wu Y et al. *JAMA Network Open.*

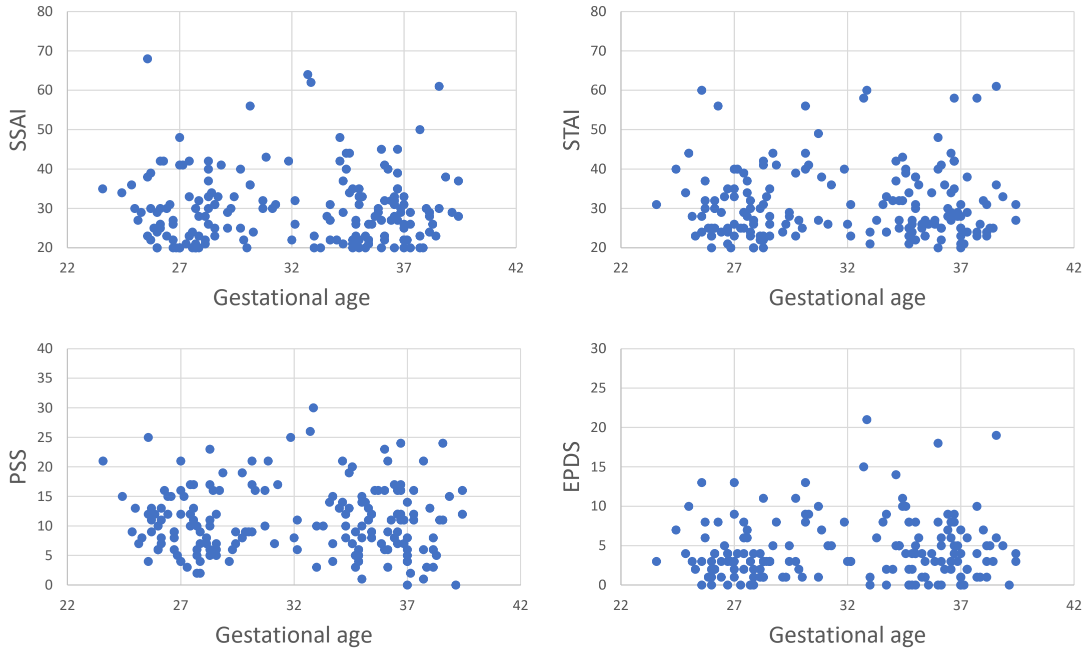

eFigure 2. Prenatal Maternal State Anxiety (SSAI), Trait Anxiety (STAI), Stress (PSS), and Depression (EPDS) Scores Across Gestational Age (Weeks)

© 2022 Wu Y et al. *JAMA Network Open.*

eTable 1. Infant Neurodevelopmental Outcomes With One and More Positive Maternal Distress Measures (SSAI, STAI, PSS, and/or EPDS)

**Test domain (mean)**	1 measure (12 subjects)	2 measures (8 subjects)	3 measures (9 subjects)	4 measures (6 subjects)	P value
**BSID-III**					
Cognitive	108	94	99	104	0.67
Language	89	94	108	110	0.21
Motor	107	95	102	104	0.19
Social-emotional	112	112	115	116	0.98
Adaptive	107	95	101	116	0.05
**ITSEA**					
Externalizing	46	54	52	49	0.26
Internalizing	47	47	56	43	0.13
Dysregulation	44	42	49	35	0.22
Competence	49	47	51	53	0.79

Abbreviations: SSAI, Spielberger State Anxiety Inventory; STAI, Spielberger Trait Anxiety Inventory; PSS, Perceived Stress Scale; EPDS, Edinburgh Postnatal Depression Scale; BSID-III, Bayley Scales of Infant and Toddler Development, third edition; ITSEA, Infant-Toddler Social and Emotional Assessment.

P values for difference between subjects with one and more maternal psychological distress based on ANCOVA, controlling for maternal education, maternal employment, total stress scale from Parenting Stress Index-Short Form at 18-month testing, and neurodevelopmental assessment during COVID-19 pandemic (yes or no).

© 2022 Wu Y et al. *JAMA Network Open.*

eTable 2. Causal Mediation Analysis of the Relationship Between Prenatal Maternal Stress and 18-Month Cognitive Outcome With Fetal Brain Measure as the Mediator

Mediator	Effect Type	β (95% CI)
Left hippocampal volume	Natural Indirect Effect	-0.11 (-0.35 to -0.0002)
	Natural Direct Effect	-0.91 (-1.62 to -0.32)
	Total Effect	-1.02 (-1.73 to -0.36)
	Percentage Mediated	10.89
Right hippocampal volume	Natural Indirect Effect	-0.0002 (-0.11 to 0.09)
	Natural Direct Effect	-1.02 (-1.85 to -0.40)
	Total Effect	-1.02 (-1.89 to -0.38)
	Percentage Mediated	0.02
Local gyrification index	Natural Indirect Effect	-0.03 (-0.27 to 0.03)
	Natural Direct Effect	-1.19 (-2.14 to -0.53)
	Total Effect	-1.22 (-2.26 to -0.58)
	Percentage Mediated	2.62
Sulcal depth	Natural Indirect Effect	-0.02 (-0.17 to 0.04)
	Natural Direct Effect	-1.20 (-2.01 to -0.47)
	Total Effect	-1.22 (-2.03 to -0.48)
	Percentage Mediated	1.68
Choline	Natural Indirect Effect	0.04 (-0.01 to 0.21)
	Natural Direct Effect	-0.94 (-1.69 to -0.35)
	Total Effect	-0.90 (-1.62 to -0.32)
	Percentage Mediated	-4.46
Choline	Natural Indirect Effect	0.038 (-0.02 to 0.20)
	Natural Direct Effect	-0.92 (-1.62 to -0.29)
	Total Effect	-0.88 (-1.59 to -0.27)
	Percentage Mediated	-4.26

Model adjusted for gestational age at fetal visit, maternal education, maternal employment, total stress scale from Parenting Stress Index-Short Form at 18-month testing, and neurodevelopmental assessment during COVID-19 pandemic (yes or no).
Note: 95% CI is bootstrap bias corrected 95% confidence limits

© 2022 Wu Y et al. *JAMA Network Open.*
